# Comparative analysis of rosaceous genomes and the reconstruction of a putative ancestral genome for the family

**DOI:** 10.1186/1471-2148-11-9

**Published:** 2011-01-12

**Authors:** Eudald Illa, Daniel J Sargent, Elena Lopez Girona, Jill Bushakra, Alessandro Cestaro, Ross Crowhurst, Massimo Pindo, Antonio Cabrera, Esther van der Knaap, Amy Iezzoni, Susan Gardiner, Riccardo Velasco, Pere Arús, David Chagné, Michela Troggio

**Affiliations:** 1 IRTA, Centre de Recerca en Agrigenòmica CSIC-IRTA-UAB, Carretera de Cabrils Km 2, 08348 Cabrils (Barcelona), Spain; 2 East Malling Research, New Road, East Malling, Kent ME19 6BJ UK; 3 The New Zealand Institute for Plant & Food Research Limited, Palmerston North Research Centre, Private Bag 11600, Palmerston North 4442, New Zealand; 4 IASMA Research and Innovation Centre, Foundation Edmund Mach, Via E Mach 1, 38010 San Michele all'Adige (TN), Italy; 5 The New Zealand Institute for Plant & Food Research Limited, Mt Albert Research Centre, Private Bag 92169, Auckland 1142, New Zealand; 6 Department of Horticulture and Crop Science, The Ohio State University/Ohio Agricultural Research and Development Center, Wooster OH 44691, USA; 7 Department of Horticulture, Michigan State University, East Lansing MI 48824, USA

## Abstract

**Background:**

Comparative genome mapping studies in Rosaceae have been conducted until now by aligning genetic maps within the same genus, or closely related genera and using a limited number of common markers. The growing body of genomics resources and sequence data for both *Prunus *and *Fragaria *permits detailed comparisons between these genera and the recently released *Malus × domestica *genome sequence.

**Results:**

We generated a comparative analysis using 806 molecular markers that are anchored genetically to the *Prunus *and/or *Fragaria *reference maps, and physically to the *Malus *genome sequence. Markers in common for *Malus *and *Prunus*, and *Malus *and *Fragaria*, respectively were 784 and 148. The correspondence between marker positions was high and conserved syntenic blocks were identified among the three genera in the Rosaceae. We reconstructed a proposed ancestral genome for the Rosaceae.

**Conclusions:**

A genome containing nine chromosomes is the most likely candidate for the ancestral Rosaceae progenitor. The number of chromosomal translocations observed between the three genera investigated was low. However, the number of inversions identified among *Malus *and *Prunus *was much higher than any reported genome comparisons in plants, suggesting that small inversions have played an important role in the evolution of these two genera or of the Rosaceae.

## Background

Economically, the Rosaceae is one of the most important plant families [1] comprising some 90 genera with over 3000 distinct species having chromosome numbers ranging from *x *= 7 to *x *= 17 [2]. Four sub-families are distinguished on the basis of fruit types: the Maloideae (including *Malus*, and *Pyrus*); the Prunoideae (*Prunus *and other stone fruit and almonds); the Rosoideae (*Fragaria*, *Rubus*, and *Rosa*); and the Spiraeoideae (containing many ornamental species, including *Physocarpus*) [3]. A recent phylogenetic treatment of the Rosaceae based on DNA sequence data of nuclear and chloroplast genomic regions reclassified the genus into three sub-families (the Dryadoideae, the Rosoideae and the Spiraeoideae), each containing a number of distinct supertribes [2]. *Prunus *and *Malus *are included in the Spiraeoideae, supertribe Amygdaleae and Pyrodae (tribe Pyrinae) respectively, whilst *Fragaria *is included in the Rosoideae, supertribe Rosodae (tribe Fragariinae). It has been postulated that the poor phylogenetic resolution along the backbone of the Rosaceae phylogenetic tree suggests a rapid evolutionary radiation of lineages within the family, corresponding to a relatively recent divergence of the genera [2]. Possibly because of the rapid evolution, members of the Rosaceae display remarkable phenotypic diversity, with common morphological synapomorphies not readily identifiable. Indeed, plant habit, chromosome number, and fruit type have all evolved independently on more than one occasion within the family [2,4,5]. A better understanding of how the phenotypic diversity within the Rosaceae arose would provide an insight into how evolution can lead rapidly to diversification.

Comparative mapping has been carried out in a number of economically important plant families including the Poaceae, Solanaceae, Brassicaceae and Fabaceae [6-10]. In the Poaceae, marker order is highly conserved within syntenic 'genome blocks' between genera [8]. However, despite the conservation of syntenic blocks, grass lineages may rapidly evolve, with high rates of chromosomal 'reshuffling' observed between the rye and wheat genomes [11]. Using restriction fragment length polymorphisms (RFLPs), Bonierbale *et al. *[12] studied the conservation of synteny between potato (*Solanum tuberosum*) and tomato (*S. lycopersicum *syn. *Lycopersicon esculentum*) and found remarkable conservation of genome structure, with only a few regions where paracentric chromosomal rearrangements could be identified. More recently, Wu and Tanksley [13] reported a higher frequency of inversions than translocations among the genomes of different genera of the Solanaceae.

In the Brassicaceae, almost complete genome colinearity between *Arabidopsis thaliana *and *Capsella rubella *has been observed, with gene repertoire, order and orientation highly conserved [14] and likewise, soybean linkage group A2 was shown to be conserved over its entire length with *Arabidopsis *chromosome I, with just 3 rearrangements identified between the chromosomes of the two species [15]. However, between *Arabidopsis *and *Brassica oleracea*, rates of chromosomal rearrangements were shown to be much higher [16]. Between dicotyledenous families, comparisons have been performed between much wider evolutionary distances, for example between *Prunus *and *Arabidopsis *[17-19], but only fragmentary patterns of conserved synteny have been observed. However, intrafamilial studies have shown that genome evolution within a family usually proceeds through whole-scale inversions and translocations between chromosomes, meaning regions in which marker order is highly conserved can be identified between genera that diverged millions of years ago, and thus information on genes within conserved genome blocks of one genus can inform studies in other genera within a family.

Comparative genome studies in the Rosaceae have so far been based on the alignment of genetic maps within the same genus, or amongst closely related genera, using small sets of orthologous markers. These studies showed that the genomes of *Prunus *species are essentially collinear, for example in peach and apricot [20,21], and peach and sweet cherry [22,23]. Similarly, within the Pyrinae tribe, the genomes of *Malus*, *Pyrus *and *Eriobotrya *were shown to be highly collinear [24-26]. Only a few studies investigated genome comparisons across Rosaceae tribes or subfamilies. Dirlewanger *et al. *[27] compared *Malus *and *Prunus *and found strong evidence that single linkage groups in the diploid *Prunus *were homologous to two distinct homeologous linkage groups in the amphitetraploid genome of *Malus*. Vilanova *et al. *[28] compared the diploid reference linkage maps for *Prunus *(T×E; almond 'Texas' × peach 'Earlygold') and *Fragaria *(FV×FN; *F. vesca '*815' × *F. nubicola *'601') and they identified numerous chromosomal translocations and rearrangements that occurred in the 29 million years since the genera diverged from a common ancestor. They also found clear cases of conservation of chromosomal synteny, and reconstructed a hypothetical ancestral Rosaceae genome composed of nine chromosomes.

Whole genome sequencing using next generation technologies has now become accessible to the broad scientific community. In the Rosaceae, the genome of *Malus × domestica *was recently sequenced using a whole genome shotgun approach [29]. The analysis of the draft sequence of 'Golden Delicious' is consistent with a putative nine chromosome diploid ancestor for the genus [28]. Although there are no published genome sequences that would permit direct comparisons of the *M. × domestica *genome and those of other genera of the Rosaceae, there are a large number of genetic markers available for Rosaceous species. Recently, Cabrera *et al. *[30] reported the development of 857 Rosaceous Conserved Orthologous Set (RosCOS), of which 613 were mapped on the T×E *Prunus *reference map. Because of the conserved nucleotide sequence and low or single copy presence across species, the COS sequences are particularly useful for comparative genome studies between related species [30]. The RosCOS set demonstrated extensive conservation of synteny between poplar and *Prunus*, members of two different plant families in the eurosid clade [30]. Also a significant fraction of the RosCOS markers were transferable to the FV×FN *Fragaria *reference population.

In this paper, we compared the apple genome sequence with conserved molecular markers previously mapped in *Prunus *and *Fragaria *[28,30,31], along with an additional set of markers developed for the purpose. The goal was to derive at the ancestral genome structure and organisation of species within the Rosaceae family and to study the genome evolution of the economically relevant *Fragaria*, *Malus *and *Prunus*. The locations of molecular markers on the *Prunus *and *Fragaria *genomes were determined through bin mapping [32,33], and their physical positions through analysis of the *Malus × domestica '*Golden Delicious' genome [29]. The comparisons identified syntenic blocks common among the genomes of the three genera as well as within the polyploid *Malus *genome. The findings allowed us to hypothesize about genome evolution within Rosaceae, and to reconstruct its ancestral genome for the family.

## Results

### Mapping of markers in the *Prunus *T×E bin set

A set of transferable expressed sequence tag (EST)-derived markers were developed in this study from consensus *Prunus-Malus *sequences by aligning all available *Prunus *EST sequences (75,404; peach EST database, [34]) to 1,262 *Malus *'Golden Delicious' gene sequences with known locations on the *M. × domestica *consensus reference map. Of the 155 novel EST-derived markers screened by PCR over the parents of the T×E population, 126 (81.2%) amplified a single band, five (3.2%) amplified a length polymorphism between the parents of the bin set that was scored by agarose gel electrophoresis, 20 (12.9%) gave no amplification product, and the remaining four (2.4%) produced multiple fragments. Of the 126 single band markers, those that produced amplicons between 100 and 300 bp (107) were analysed by high resolution melting (HRM). In total, 60 exhibited a clear segregation pattern, enabling their localization on T×E bin map, 17 were monomorphic, and 30 revealed an ambiguous pattern. The 19 PCR marker products larger than 300 bp, together with the products of 30 markers that produced ambiguous segregation patterns following HRM analysis, were sequenced directly, enabling a further 29 polymorphic markers to be mapped. Thus in total, 94 of the 155 (60.7%) new EST-derived markers were located on the *Prunus *reference map (Table S1 in Additional File 1). Their distribution ranged from one to six markers per bin, and the novel markers were evenly distributed over the eight linkage groups of the T×E map. Marker MDP0000144421 revealed a new mapping bin (7:34), however its addition did not increase the coverage of the T×E map.

### Mapping of markers in *Fragaria *FV×FN bin set

A total of 126 (23%) RosCOS markers bin mapped in *Prunus *by Cabrera *et al. *[30] were located to defined mapping bins on the FV×FN map. Additionally, results from 111 markers previously mapped in *Fragaria, Prunus*, or in *Malus *by Vilanova *et al. *and Sargent *et al. *[28,35] were included in the analysis. Markers were evenly distributed across the seven FV×FN linkage groups, with 1 - 15 markers located to each bin (Table S2 in Additional File 2).

### Marker anchoring to the *Malus × domestica *'Golden Delicious' genome sequence assembly and definition of syntenic blocks

**Figure 1:**
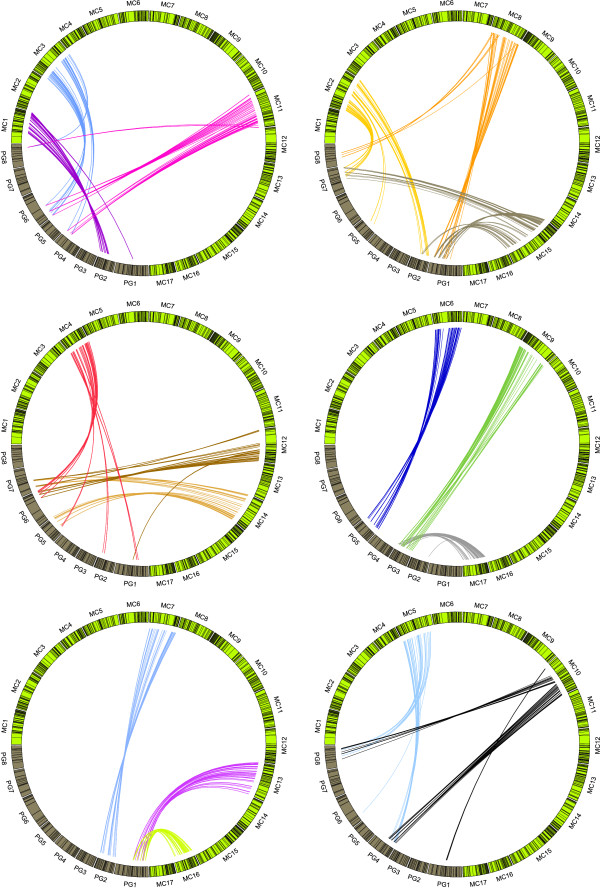
**Comparative analysis of *Malus *and *Prunus *genomes**. Comparison between *Malus *genome sequence (MC1 to MC17) and TxE *Prunus *reference map (PG1 to PG8). In the figure, only syntenic markers have been included.

**Figure 2:**
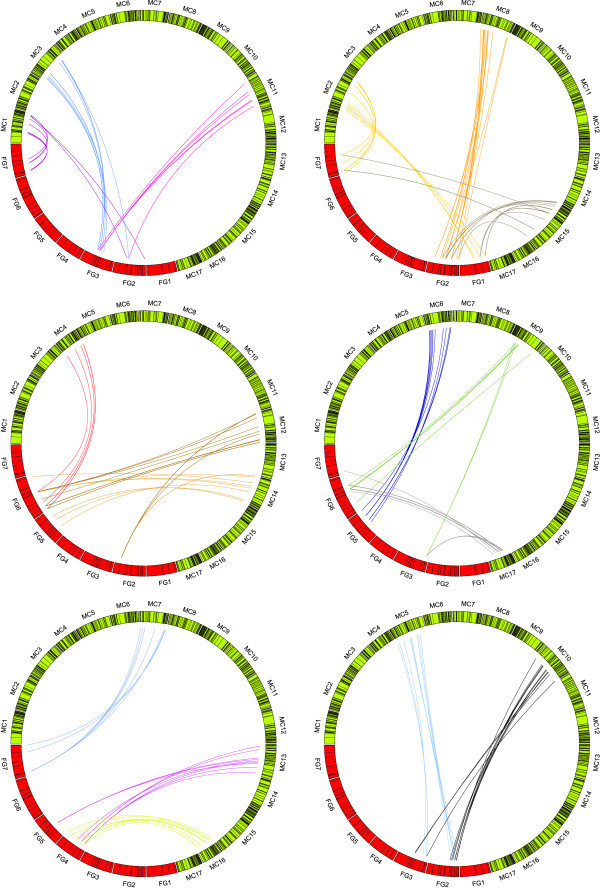
**Comparative analysis of *Malus *and *Fragaria *genomes**. Comparison between *Malus *genome sequence (MC1 to MC17) and diploid *Fragaria *FV×FN reference map (FG1 to FG7). In the figure, only syntenic markers have been included.

The sequences of 1,473 markers (572 SSRs, 117 RFLPs, 235 EST-derived markers, and 549 RosCOS), 1321 positioned in *Prunus*, and 251 in *Fragaria*, were used as queries for GMAP and BLASTN. Of these, 1,013 markers (214 SSRs, 72 RFLPs, 182 EST-derived markers and 545 RosCOS) showed a high level of DNA sequence conservation with apple, allowing them to be located to precise positions on the *Malus *chromosomes using GMAP (Table S3 in Additional File 3). In general, a single locus identified in *Prunus *or *Fragaria *detected in *Malus *either one locus, or two distinct homeologues. Following the criteria described in Materials and Methods, 806 markers were retained for syntenic analysis. Of these, 784 were common between *Malus *and *Prunus*, whilst 148 were common between *Malus *and *Fragaria *and 129 were common to all three genomes (Table 1). Figures 1 and 2 show the relationships between the 806 markers visualized using the Circos program [36]. Figure 1 shows the conservation of synteny between *Malus *and *Prunus*, Figure 2 the conservation of synteny between *Malus *and *Fragaria*, and Figure S1 in Additional File 4 shows the conservation of synteny between *Prunus *and *Fragaria*.

**Table 1:** *Prunus *and *Fragaria *mapped markers used for synteny comparison with the *Malus *genome

Marker type	RosCOS	EST	RFLP	SSR	Total^†^
*Prunus*	439	191	49	105	784 (988)

*Fragaria*	90	25	19	14	148 (164)

Prunus-Fragaria*	90	20	19	-	129

**Total unique syntenic markers**					806

*Only markers that were also present in *Malus *genome sequences were included in the *Fragaria-Prunus *comparison.

***^† ^***In parentheses, corresponding loci in the *Malus *whole genome sequence.

The assignment of collinearity of regions of *Malus *chromosomes (referred to hereafter as MC) with *Prunus *and *Fragaria *was based on the number of reciprocal translocations or fission/fusion events (which we will refer collectively as translocations) and the number of inversions which explain the current marker order in *Malus *(Table 2). When considering only the common set of 129 syntenic markers, the number of inversions was three times higher between *Fragaria *and *Malus *than between *Prunus *and *Malus*. At least 45 inversions were needed to place the markers in the same order between *Fragaria *and *Malus*, and 14 between *Prunus *and *Malus*. When compared with syntenic regions of both *Fragaria *and *Prunus*, MC11 and MC7 were characterized by complete collinearity (lack of inversions), based on markers in common across the three genera. When the 784 markers shared only between *Malus *and *Prunus *were considered, the number of inversions reached 65 across all MC, almost five times that found with the markers in common across the three genera (Figure S2 in Additional File 5). However, for MC7 and MC11 the numbers of rearrangements observed remained low, with one inversion assigned to each of the two *Malus *chromosomes. *Malus *chromosome MC8 was the most rearranged with at least eight inversions required to explain the differences in marker order between *Malus *and *Prunus*, and seven between *Malus *and *Fragaria*. The total number of translocations between *Fragaria *and *Malus *(30) was also higher than that estimated for the *Prunus-Malus *comparison. Certain *Malus *chromosomes, such as MC2, 4, 12 and 15 were highly rearranged between *Malus *and the two diploid genomes, with two or three translocations per chromosome per species, whereas others (MC7 in the comparison with *Fragaria *and MC6, 7, 9, 13, 16 and 17 in that with *Prunus*) originated each from a single ancestral DNA fragment. The same translocations were found for *Malus-Prunus *when using markers shared between the three genera or the complete set of anchor markers.

**Table 2:** Translocations and inversion events hypothesized to occur in *Prunus *and *Fragaria*, compared with *Malus*

*Malus *chromosome	*Prunus*				*Fragaria*	
	
	All markers		**Common markers**^**†**^		**Common markers**^**†**^	
	
	Translocations*	Inversions	Translocations*	Inversions	Translocations*	Inversions
MC1	1	6	1	1	1	2

MC2	2	2	2	0	3	3

MC3	2	0	2	0	2	4

MC4	3	3	3	1	3	2

MC5	1	2	1	0	1	1

MC6	0	9	0	3	1	4

MC7	0	1	0	0	0	0

MC8	1	8	1	4	2	7

MC9	0	7	0	0	3	2

MC10	2	3	2	1	2	3

MC11	2	1	2	0	1	0

MC12	2	2	2	1	3	3

MC13	0	4	0	0	1	4

MC14	1	4	1	1	2	1

MC15	2	8	2	2	3	5

MC16	0	4	0	0	1	3

MC17	0	1	0	0	1	1

**Total**	19	65	19	14	30	45

*Reciprocal translocations and fission-fusions

***^†^***The set of 129 markers in common among the three genomes

**Figure 3:**
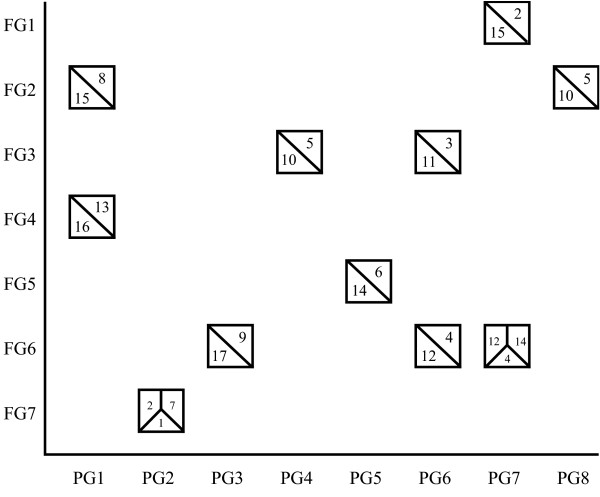
**The major syntenic relationships observed among the *Fragaria*, *Prunus *and *Malus *genomes**. The major syntenic relationships observed among the three genomes were revealed through a comparison of 806 genetic markers. *Fragaria *groups (FG1-FG7) are indicated along the *x*-axis of the graph, *Prunus *groups (PG1-PG8) along the *y*-axis, and *Malus *groups are represented by boxes plotted against the two axes. Numbers within the boxes representing *Malus *indicate the chromosomes having syntenic relationships.

The locations of markers revealed patterns of conservation of synteny across the genomes of the three genera, as summarized in Figure 3. Within the *Malus *genome, homeologous chromosomes were observed, which corresponded to those identified by Velasco *et al. *[29] on the basis of whole genome sequence information and reported earlier by Celton *et al. *[25] on the basis of SSR mapping. Four fully homeologous pairs of chromosomes were revealed: *Malus *chromosomes 3 and 11; 5 and 10; 9 and 17; and 13 and 16. More complex relationships between chromosomes 4, 6, 12 and 14; chromosomes 1, 2 and 7; and chromosomes 2, 8 and 15 were evident. Among genera, eleven major syntenic blocks were common to all three genomes and, in some cases, conservation of synteny was revealed across whole chromosomes. Amongst these, homeologous groups MC13 and MC16 were syntenic to the top of *Prunus *linkage group (PG)1 and *Fragaria *linkage group (FG)4, and the top of *Malus *homeologues MC5 and MC10 corresponded to PG4 and FG3.

### Reconstructing the ancestral genome of the Rosaceae

**Figure 4:**
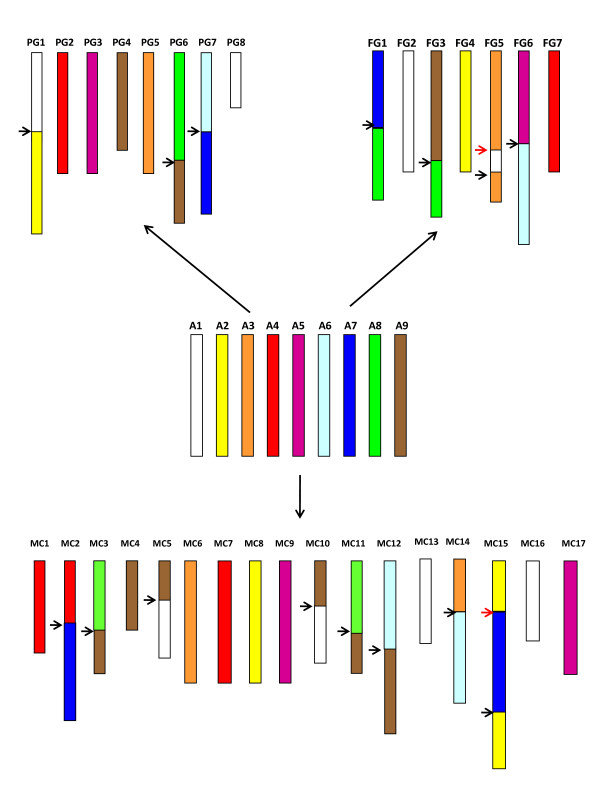
**Reconstruction of a hypothetical ancestral Rosaceae genome**. Syntenic regions among the genomes were elucidated from the positions of 129 orthologous markers shared by all three genomes. The hypothetical ancestral genome contains nine chromosomes numbered Ancestral 1 (A1) - A9. Sections of the chromosomes of *Malus *and the linkage groups of *Fragaria *and *Prunus *are coloured according to the hypothetical ancestral chromosomes. Breakpoints indicating chromosomal fusion-fission events or reciprocal translocations correspond to arrows between coloured syntenic blocks. Red arrows indicate the positions of major inversions that can be predicted based on the fusion-fission or translocations detected. Extant chromosome/linkage group lengths assume that all nine hypothetical ancestral chromosomes were of the same length.

A total of 129 markers that mapped to syntenic blocks common to the marker datasets of the three genera were used to develop a model for the ancestral genome of the Rosaceae. The conformation of syntenic blocks shared among the *Fragaria*, *Malus *and *Prunus *genomes was used to model a hypothetical ancestral genome with nine chromosomes (Figure 4). Starting from the ancestral genome and considering only translocation events, the extant *Prunus *linkage groups (*x *= 8) would have experienced three translocation events, and the *Fragaria *linkage groups four translocation events, whilst for the *Malus *chromosomes (*x *= 17), seven translocation events, two of which (a fusion of two major fragments of the two ancestral chromosomes A1 and A9 to form MC5 and MC10 and of A8 and A9 to form MC3 and MC11) preceded the whole genome duplication in apple. Figure 4 depicts the genomes of *Fragaria*, *Malus *and *Prunus *showing the positions of major chromosomal translocations necessary to create their extant genomes starting from the putative nine chromosomes of the ancestral genome. Chromosome/linkage group lengths are approximated relative to the lengths of the hypothetical ancestral chromosomes that we assumed to have a similar length. Colouring of the ancestral chromosomes follows Vilanova *et al. *[28].

## Discussion

We have conducted a comparative analysis between sequence-characterised molecular markers genetically anchored to the *Prunus *and *Fragaria *reference linkage maps and the recently published whole genome sequence of *Malus × domestica *[29]. In total, 784 *Prunus *markers meeting the conservation of synteny criteria described in Material and Methods corresponded to 988 *Malus *loci. The density distribution of these markers was higher than in previous studies involving other families (one marker every 0.65 cM in *Prunus *corresponding to one locus every 0.76 Mb in *Malus*, compared to one marker every 1.6 cM in a comparative analysis between *B. napus *and *A. thaliana*, and one marker every 6.9 cM in a comparison between *Capsicum annuum *and *S. lycopersicum *[10,37-42]. The relationships between *Fragaria *and *Malus *were based on fewer markers, which revealed 164 loci on the *Malus *genome sequence (one marker every 4.24 cM in *Fragaria *corresponding to one locus every 3.64 Mb in *Malus*). These values, despite being lower than for *Prunus*, were still within the range of densities employed for genome comparisons based on linkage maps in species of other families.

The RosCOS markers showed the highest level of transferability among genomes, with only 4 out of 549 sequences not found in the *Malus *genome sequence. This was expected because RosCOS markers have been developed from genes highly conserved across genomes that are present as low or single copy genes in *Arabidopsis *[30]. Transferability from species to species of EST-derived markers was also high, with 78% of those tested matching one or two loci in the *Malus *genome. A similar value was also observed for RFLPs generated from cDNA libraries. In contrast, SSRs derived from genomic regions were less conserved among the three genomes, as reported in previous studies [43]. While SSRs have been shown to be transferable between genera in a family, the evolutionary relationship between target and source genus plays an important role [44]. For example, a higher level of SSR transferability between *Prunus *and *Malus *compared with *Fragaria *and *Prunus *was expected, since *Prunus *is phylogenetically closer to *Malus *than to *Fragaria *[2]. The results presented in this paper indicate that the majority of genes are conserved among the three genera investigated, however that significantly higher divergence was observed in non-coding regions of the genome than coding. Correspondence among marker/gene positions in the three genomes was high in all pair-wise comparisons, with clear conserved syntenic blocks identified between genera. The 129 markers present in syntenic blocks and common to all three genomes allowed us to propose a hypothetical ancestral genome for the Rosaceae consisting of nine chromosomes. Our study considered nearly twice the number of markers used by Vilanova *et al. *[28] in an analysis of the FV×FN and T×E linkage maps. The additional markers revealed an additional translocation event between the hypothetical ancestral chromosomes A1 and A3 to create the extant *Fragaria *linkage group FG5. The same set of marker data fully support the findings of Velasco *et al. *[29], who postulated an ancestral diploid progenitor for *Malus *with a genome containing a chromosome complement of *x *= 9. Our data also revealed a fusion/fission event between A1 and A9 during the formation of MC5 and MC10. This occurred before the major whole genome duplication of *Malus *and thus was not identified in the analysis of the *Malus *genome alone.

Whilst an early hypothesis as to the origin of *Malus *implied a wide hybridisation between an ancestral amygdaloid (*x *= 8) and an ancestral spiraeoid (*x *= 9) [45], other data suggest that *Malus *may have arisen due to a polyploidization of a spiraeoid species [46,47]. The more recent hypothesis is supported by molecular phylogenetic analyses based on various nuclear encoded genes [48], and by the comparison between *Malus *DNA sequence data and that of various Rosaceae species [29], which showed that *Gillenia *(*x *= 9) was the closest extant diploid genus to both homeologous genomes of *Malus*. Our results indicate that from the four pairs of homeologous chromosomes entirely conserved in apple, two pairs, MC3-MC11 and MC5-MC10, were assembled from unique major fragments of two ancestral chromosomes. This suggests that the translocations that produced them pre-date the duplication within the apple genome, and were common to the ancestor(s) of apple. The fact that none of these corresponded to rearrangements found in *Prunus *indicates that its genome is an unlikely ancestor of apple. In all, these results support the hypothesis that the two genomes that constitute apple were similar, however distant from *Prunus*, in agreement with the hypothesis of a possible origin of apple as an autotetraploid from a *x *= 9 genome [29]. Hence, evidence from both previous phylogenetic analyses [48] and the comparative mapping data presented here supports the hypothesis that a genome containing nine chromosomes is the most likely most recent ancestral progenitor of the Rosaceae.

Upon pair-wise comparison of each of the three genomes with the ancestral Rosaceae genome (Figure 4), we found that the number of major chromosomal translocations separating them was four, three and eight respectively, which is consistent with a relatively uniform rate of translocation, considering that the apple genome is tetraploid and thus the average number of estimated translocations per genome is four. When using the set of 129 markers common to all three genera, including all rearrangements (major and minor, inversions and translocations) we found that *Malus *and *Prunus *differed by 19 translocations and 14 inversions (an average of 9.5 translocations and seven inversions per diploid genome) and *Malus *and *Fragaria *by 30 translocations and 45 inversions (15 and 22.5). Nine translocations and 27 inversions were estimated when comparing *Fragaria *and *Prunus *[28]. The total number of rearrangements is consistent with genome divergence [2], with less (an average of 16.5) between *Malus *and *Prunus *and more in the comparisons involving *Fragaria *(36-37.5). Our data suggest that the number of inversions (seven for *Malus *and *Prunus *and 22 and 27 for the comparisons involving *Fragaria*) better estimate the evolutionary distance between genera than the number of translocations, as the comparison of inversions between *Fragaria *and *Prunus *yields a smaller number (9) than predicted based on the other two comparisons.

When we compared the whole set of 784 markers of *Prunus *to the *Malus *genome, the number of translocations identified was the same as when using the 129 markers in common, however the number of inversions was 65, almost five times higher than that estimated with fewer markers. As expected, most of these inversions were found in relatively small DNA fragments, with 71% (46) of them concerning apple genomic fragments ≤ 7.5 Mbp (1% of the *Malus *genome). Other explanations could account for errors in this estimate, such as wrongly-oriented scaffolds in the whole genome apple sequence, or markers misplaced on the linkage maps. It seems however unlikely that they can account for the majority of the inversions observed. When we re-examined our data and discarded 20 markers that, if misplaced would have generated one or more spurious inversions each, we found a more conservative estimate of 43 inversions (more than three times that of the common set of markers), with 58% of them (25) involving apple DNA fragments ≤ 7.5 Mbp, still a very large number of inversions. We attribute this high frequency of inversions to the fact that our comparison is based on an unusually high average density of markers, allowing the identification of much smaller inverted regions compared with other plant families. Paterson *et al. *[49] did not find such high numbers of inversions in the comparison between the genome sequences of *Sorghum bicolor *and *Oryza sativa *and another comparison between a map of *B. napus *with high marker density (0.63 markers/cM) with the genome of *Arabidopsis *(3.67 markers/Mb) allowed the detection of only eight inversions [39]. A pattern of chromosomal evolution involving many small inversions has been found in *Drosophila *[50], suggesting that inversions may be an important driving force in the evolution of Rosaceae or at least of the genera investigated, being partly responsible for the high level of phenotypic diversity observed among members of this family.

Based on data from the comparison of genomes of various plant families, Paterson *et al. *[51] showed that the difference between plant genomes consist of a finite number of chromosomal rearrangements, and estimated average frequency of 0.14 chromosomal rearrangements per Myr of divergence time from a common ancestor. From our data, this may be a reasonable approximation for a low/medium resolution level in terms of anchor points per Mb of the genome; however, in higher resolution analyses, small inversions may completely change the picture, leading to a variable number of breakpoints depending on the particular history of the genera being compared.

## Conclusions

We have used markers located to the genomes of *Fragaria*, *Malus *and *Prunus *to conduct the first detailed family-wide comparative analysis of the Rosaceae. The high density distribution of markers analysed throughout the genomes of the three genera permitted a detailed study of the conservation of synteny, comparable to those performed in other plant families, such as the Solanaceae. Clear syntenic blocks that were conserved across the family were identified, and a hypothetical ancestral genome for the Rosaceae has been reconstructed using 129 common markers. The hypothetical ancestral genome contained nine chromosomes and was comparable to hypotheses proposed in previous studies of *Malus, Fragaria *and *Prunus *[28]. Our study revealed novel syntenic relationships that were not resolved in previous investigations. The number of inversions identified among *Malus *and *Prunus*, based on 784 markers was much higher than that estimated with fewer markers, and higher than any reported genome comparisons in plants, suggesting that small inversions have played an important role in the evolution of these two genera or of the Rosaceae.

## Methods

### Development and bin mapping of novel EST-derived markers in *Prunus*

A total of 155 primer pairs were designed from consensus *Prunus-Malus *sequences for a set of genes that were well-distributed throughout the 17 *Malus *linkage groups, using PRIMER 3 [52] with default parameters set to give an expected product size from 150-300 bp with a small number of exceptions. Primers were tested for amplification in the parents of the *Prunus *reference mapping population (T×E) following Troggio *et al. *[53].

All primer pairs amplifying a single product of 300 bp or less were used to test for polymorphism between the parental genotypes and the six plants of the T×E bin set [32] using HRM analysis methodology [54] as follows. PCR was performed in a total volume of 10 μl containing 20 ng of template DNA, 2.5 mM MgCl_2_, 300 nM forward and reverse primers and 1× HRM master mix (Roche Applied Science). Both PCR and HRM were performed on a Roche LightCycler^® ^480 (Roche Applied Science). The PCR parameters used were an initial denaturation step of 95°C for 10 min, followed by 45 cycles of 95°C for 10 s, 57°C for 15 s, and 72°C for 15 s. Following amplification, the samples were heated to 95°C for 1 min and then cooled to 40°C for 1 min. Melting curves were generated with continuous fluorescence acquisition during a final ramp from 65°C to 95°C at 1.1°C/s and the resultant fluorescence data were processed using the LightCycler480^® ^software (version 1.5.0.39; Roche Applied Science).

Amplicons greater than 300 bp in length and those that gave ambiguous results following HRM analysis were sequenced from the parental genotypes and the bin set following the methods of Troggio *et al. *[53]. Sequence data were aligned using SEQUENCER v4.8 (Gene Codes Corporation; Ann Arbor, MI, USA), all markers were scored in the bin set and their locations added to the *Prunus *reference bin map.

### Bin mapping of RosCOS markers in *Fragaria*

Primer pairs for the 549 RosCOS markers [30] that were successfully anchored to the *M. × domestica *genome sequence were screened across the parents of the diploid *Fragaria *mapping population FV×FN [28,33] following the methods of Sargent *et al. *[55]. Markers that amplified a discrete PCR product were scored in the FV×FN bin set [33] either following agarose gel electrophoresis, or capillary electrophoresis as described in Sargent *et al. *[55]. Primers which did not reveal visible polymorphisms were extended with a 5' M13F sequence on the forward primer and products were sequenced and scored for single nucleotide polymorphisms (SNPs) as described by Sargent *et al. *[56].

### Marker anchoring to the *Malus × domestica '*Golden Delicious' genome sequence assembly

The high quality draft genome sequence of *M. × domestica '*Golden Delicious' [29] was used to locate markers bin mapped in *Fragaria *and *Prunus*, to the *Malus *genome. Marker sequences were used as queries for GMAP [57] and as a validation step, BLASTN, using a cut off E-value of 1e-15.

### Identification of chromosomal rearrangements and determination of syntenic blocks among *Malus*, *Prunus *and *Fragaria*

Markers showing a high degree of DNA sequence conservation in apple (BLAST E-value < e-15) and that were located to the 'Golden Delicious' genome with GMAP, together with the novel EST-derived mapped markers, were used for the conservation of synteny analysis. Syntenic blocks among *Malus*, *Prunus *and *Fragaria *were defined by the following criteria: a syntenic block should contain a minimum of five (*Prunus*) or three (*Fragaria*) homologous marker loci mapping to one or two contiguous bins in *Prunus *and *Fragaria*, and located within 3.5 Mbp from each other on the *Malus *genome. Markers originally mapped in the full populations of either *Prunus *or *Fragaria*, were converted to bin positions before the analysis. To visualise the relationships between the genomes of the three genera, the Circos software package was used [36]. As input data for Circos, the physical positions of markers on the *Malus *genome were divided by 4 × 10^5 ^to obtain a genetic distance equivalent to marker linkage values of the *Malus *consensus map [29]. For the *Fragaria *and *Prunus *bin sets, a genetic distance corresponding to the mid-point of each mapping bin was considered. Comparisons between *Malus *and *Prunus*, *Malus *and *Fragaria*, and *Fragaria *and *Prunus *were then visualised, with three linkage groups represented in each ideogram. The method of Vilanova *et al. *[28] was used to estimate the number of chromosomal rearrangements which occurred among the three genomes since they diverged from a common ancestor.

### Reconstruction of a hypothetical ancestral genome for Rosaceae

The set of 129 genetic markers located on the genomes of all three genera were used to define a hypothetical ancestral genome for the Rosaceae. The positions of these markers were used to identify homeologous chromosomal regions within the *Malus *genome, and subsequently homologous chromosomal segments on the *Fragaria *and *Prunus *reference maps. We started with the chromosomal evolution hypothesis of strawberry and peach elaborated by Vilanova *et al. *[28], modified it with the new data provided by this research, and then constructed the apple chromosome complement using the more complete *Prunus*-*Malus *comparison mainly. As we intended to present a simplified view of the complexity of this comparison, we considered translocated segments as only those that involved large DNA fragments (*i.e*. that covered ≥4.41 Mbp of the apple sequence, equivalent to a 10% of the average apple chromosome 750Mbp*0.10/17 chromosomes). We only included two inversions, one in strawberry and another in apple, that were obvious consequences of the major rearrangements considered in this ancestral genome reconstruction.

## Abbreviations

RFLP: restriction fragment length polymorphism; SSR: simple sequence repeat; T×E: almond 'Texas' × peach 'Earlygold'; FV×FN: *Fragaria vesca '*815' × *Fragaria nubicola *'601'; RosCOS: Rosaceous conserved orthologous markers; EST: expressed sequence tag; HRM: high resolution melting; SNP: single nucleotide polymorphisms; MC: *Malus *chromosome; PG: *Prunus *linkage group; FG: *Fragaria *linkage group; WGD: Whole genome duplication.

## Authors' contributions

PA, MT, DJS, DC, SG and RV conceived and designed the experiment. DJS, MT, PA, EI, and DC wrote the paper with editorial comment from the other co-authors. EI, ELG, JB and MP carried out the experiments and provided interpretation of the results. AC and RC performed the bioinformatics analyses and provided interpretation of the results. ACa, EVK and AI gave access to the RosCOS before publication. All authors read and approved the final manuscript.

## Supplementary Material

Additional file 1 **Table S1, novel *Prunus *EST-derived markers**. Table S1 lists locus names, primer sequences, and the T×E bin map positions of the 155 novel EST-derived markers mapped in this work. Click here for file

Additional file 2 **Table S2, *Fragaria *genetic markers**. Table S2 lists the 237 genetic markers of the diploid *Fragaria *FV×FN reference map, along with their marker type, method of detection and the mapping bins to which they are located. Click here for file

Additional file 3 **Table S3, markers used in this work**. Table S3 lists the number and type of markers used in this work. All the available markers were tested against *Malus × domestica *genome using GMAP and BLASTN. Syntenic markers are those that fulfilled the criteria defined for conservation of synteny in the Materials and Methods section. Click here for file

Additional file 4 **Figure S1, *Prunus *and *Fragaria *map comparison**. Figure S1 shows the comparative analysis between *Prunus *(PG1 to PG8) and diploid *Fragaria *(FG1 to FG7) reference map using the Circos program. Only markers that were also present in the *Malus *genome sequences were included in the *Fragaria*-*Prunus *comparison. Click here for file

Additional file 5 **Figure S2, *Malus *and *Prunus *inversions in the *Malus *genome**. Figure S2 shows the positions in the *Malus *genome of the inversions detected between *Malus *and *Prunus *using the complete set of 784 anchor markers in *Prunus*. Apple chromosomes are divided in fractions of 10 Mb. Arrows indicate the presence of a predicted inversion. Click here for file
