# Supplementary material for: Comparative analysis of rosaceous genomes and the reconstruction of a putative ancestral genome for the family
Source: BMC Evol Biol. 2011 Jan 12;11:9. doi: 10.1186/1471-2148-11-9 (PMC3033827; doi:10.1186/1471-2148-11-9)
Supplement: Additional file 1 — Table S1, novel Prunus EST-derived markers. Table S1 lists locus names, primer sequences, and the T×E bin map positions of the 155 novel EST-derived markers mapped in this work. [file 1471-2148-11-9-S1.DOC]

## Table S1. Locus names, primer sequences, and the T×E bin map positions of the 155 novel EST-derived markers mapped in this investigation.

| ***Prunus* marker** | **Forward primer 5' to 3'** | **Reverse primer 5' to 3'** | **Amplification / polymorphism** | **Detection method** | **T×E bin map position** |
| --- | --- | --- | --- | --- | --- |
| MDP0000029168 | TGAGTTTCGGTGAGAGCAAG | GTTTTCTCGGTCGTTTTTCG | Yes / Yes | HRM | 6:84 |
| MDP0000047001 | GATGGCAAGGTGGAAGCATA | CGCTCCAAATATCTGAGGAA | No / - | - | - |
| MDP0000069231 | TGGTGTCTTTGACAACCAGC | ATTAGGCGGCGGTAACCTT | Yes / Yes | HRM | 5:08 |
| MDP0000127923 | CATCCAGAAATTGAGCAAGG | CCGGTTGCAGTTTTAGGAAG | Yes / Yes | Sequencing | 5:21 |
| MDP0000131015 | CCCGAGTCCACGAATAAGG | GCTGTGGTTGTTGGAGAAGA | No / - | - | - |
| MDP0000131537 | GTACTGGAAGGACGACGGCT | CGGCCTTTGTTTCTCACAAT | Yes / Yes | Length size polymorphism | 3:37 |
| MDP0000131695 | TGGTGATTTTAATCCAACGC | CCACTTCTTTCATCTGCTCG | Yes / Yes | Sequencing | 3:37 - 3:49 |
| MDP0000135455 | ATGCAAAGGACCACAGTACC | CCCGGTGGGTAGTTTCCATA | Yes / No | Sequencing | - |
| MDP0000135672 | GTTGTCGTAGGCGACAGCA | ACGGGATGAGGTGAAAACAT | Yes / Yes | HRM | 2:13 |
| MDP0000139971 | AATGGGGCAAAGCATCTGTA | CGGAAACTGGTGAATGCTCT | No / - | - | - |
| MDP0000141008 | AATGGGCACCGGTATGAAG | GGGAGATCCAGGATGGGTAT | Yes / Yes | HRM | 6:84 |
| MDP0000142642 | AGAGCGGGTCCTTCTCGTA | CTCTGCTTTGCCTCAACCTC | Yes / Yes | HRM | 6:45 - 6:49 |
| MDP0000144421 | ACGAGGGAATCTGAAGGTCA | TCCGACGTGGTTGGAAGATA | Yes / Yes | HRM | 7:35 |
| MDP0000144996 | CTGGTGGGCTCTTGGTATTC | GCAAAGAGAGGCACATCGAG | No / - | - | - |
| MDP0000146698 | AGCACCCTGTTGGCTTACAA | TGCTTCCTTGATTTCATCCC | Yes / Yes | Sequencing | 3:49 |
| MDP0000148778 | CGCTTGTTAGAGTTGCTGGA | TTTACAGGCACAACAATCGG | No / - | - |  |
| MDP0000148820 | CTATCTGCTGGTGGAATGGA | CTCTGGCTTGCCACTAATGA | Yes / Yes | Sequencing | 6:25 |
| MDP0000151762 | AGCTATGGTGAACATGTGGG | TTCATTTCGCAAGAAAGCCT | Yes / Yes | Sequencing | 5:21 |
| MDP0000153584 | AAATTGGCCAAGAAAGATGA | CAGTCCCGACCAAGGACTC | Yes / Yes | HRM | 3:12 |
| MDP0000154828 | TATCTCATGACGGGTAGGGG | ACCCGATTTCGTGATGCTAA | Yes / No | Sequencing | - |
| MDP0000155799 | CCAAGGGTCCCTGATGCTAC | GTGCAGTTCTGCAGCAGGT | Yes / Yes | HRM | 5:21 |
| MDP0000162236 | CAAAGGGCAGGCGTTACAT | CGAACCACAGAGAGACGAAG | Yes / Yes | Sequencing | 5:21 |
| MDP0000170069 | GAAACCCCTACAGTTGGGAA | GATGATTATGGCAAACCCGA | Yes / Yes | HRM | 8:41 |
| MDP0000170872 | TGTGAGAGACATCCAAGGGA | ACTTCTGCATCGTCTGCAAG | Yes / No | HRM | - |
| MDP0000181224 | ACTTCACGGCCTTCTGACAT | CGGCAACCTCCTGAAGAAAT | Yes / Yes | HRM | 7:25 - 7:31 |
| MDP0000182255 | ATTACCGAGACCCCTTCCTG | TCTTTCTTCGATCAACCCCA | Yes / Yes | HRM | 2:45 |
| MDP0000182948 | GTTAGAGGGAGCTCTGGCG | GCTCCAATGCATAAAATTGC | Yes / Yes | Sequencing | 7:71 |
| MDP0000183819 | AACCGTACCCGGATCCAT | TCTCCGGCAAAGATCGAG | Yes / Yes | Sequencing | 1:28 |
| MDP0000187312 | CCCCATCAACAGACTCCACT | GGCATTCATGTTCTGGCTG | Yes / No | HRM | - |
| MDP0000188396 | TTGATATTTGGTGCCACTGA | GGGGGTTCCCAAACCTTAAA | No / - | - | - |
| MDP0000190478 | GCGAGAGGCTTTGTTGAATC | GCAGATGATGCTCTCAGTCG | No / - | - | - |
| MDP0000191034 | CTCCAGGATCAAGCTTCCTT | CTTCCTGTTCTCCTTGCAGC | Yes / Yes | HRM | 4:18 |
| MDP0000191110 | TTATGGGCGGAAGGTCAAG | CAGGTTCCTCCTCAAGTGAA | Yes / Yes | HRM | 1:34 |
| MDP0000191398 | AGGGATGGGACTTGAAGTTG | AGTCTCGCTGAGCTTGGACC | Yes / No | Sequencing | - |
| MDP0000193639 | GGCATTCTCCGACAGACATT | AGAGCCTGCAGTCGTTTCTG | Yes / Yes | HRM | 8:41 |
| MDP0000194146 | GCTGGACAGTTTGTCATGGG | TTCATCCTCAGTCACCCACA | Yes / No | Sequencing | - |
| MDP0000194340 | AGCACCAAGGTTCCAGAAGA | GAGAGGCGAGGTCATCAAAC | Yes / No | Sequencing | - |
| MDP0000195064 | CCTAAAGGTGGACGTGGATG | TGCTTGGCAACTTTGATCTG | Yes / No | Sequencing | - |
| MDP0000195885 | TCCCAGTTGTTGACTTGAGC | TGCGACCATTTCCTTAAACC | Yes / Yes | HRM | 4:18 |
| MDP0000199386 | GATCGTCTCCTGTCCCTTGC | CCATGCTTTATGCAAGCTGT | Yes / Yes | HRM | 1:14 |
| MDP0000204066 | ACGTACCACTCGCAGGTGA | GCATAAGAACGGCAGCCTC | Yes / Yes | HRM | 1:14 |
| MDP0000215372 | GACCTATGCCCAAGTTGCAG | TTGGTGACTTCGCTTGATTT | Yes / Yes | Sequencing | 6:25 |
| MDP0000217690 | AACCCCAACAAGAAGATCGG | CCCACATTTAATCCTGGGCT | Yes / Yes | Sequencing | 7:31 |
| MDP0000217948 | CTTGCTGCAATAACAGGTCA | ATTCTGAGTGGCAAAAAGCA | Yes / Yes | HRM | 3:12 |
| MDP0000220005 | GTCCTTTGCTGGGGCATC | TGTCCGGTGTTTCCTGTCTA | Yes / Yes | HRM | 8:41 |
| MDP0000221809 | GGCTTGTTTACAGGGGTCAC | CCCAGCTAGTCCATTAGCCA | Yes / Yes | HRM | 3:49 |
| MDP0000222098 | ACCGACCCTTCATCAGGAAA | AACTGCAATTGACTTCGGCT | Yes / Yes | HRM | 7:25 |
| MDP0000225700 | GATGTCTTGCGTGAATTTGC | ATTTCCAGCAAGGGTCGG | Yes / No | Sequencing | - |
| MDP0000230768 | TCAAGGCTGCTAGCTTACCC | CATGTTTTGTGACAGGACCC | Yes / Yes | HRM | 2:50 |
| MDP0000231818 | CATTTCACCACCATAAGGCA | CAACCCCAACCACCTTAATC | Yes / Yes | HRM | 4:46 |
| MDP0000233179 | GGGCACTGCATGGAAGTAGA | AATTCAAACCCAAGCAACTG | Yes / Yes | HRM | 4:18 |
| MDP0000234499 | GGAGCACACTGAATGAGCC | GGAGCTGGTGGTGTGTTACC | Yes / No | Sequencing | - |
| MDP0000235846 | CTCACCAAGTACGGGTCAGA | CGTTGCAACATACCCATCAG | No / - | - | - |
| MDP0000237026 | CAGCATGAATCCAATGTTGA | TCTTGGTCGTGGTCCTTTG | Yes / Yes | HRM | 6:39 |
| MDP0000238726 | ATAAAATGCATGCACGAGGA | CTTGCAAGCATAGGGAAGC | Yes / Yes | Sequencing | 2:08 |
| MDP0000238936 | GACCACATTGCGGATGATT | CTCCGTAAGTATCGCTTGGC | Yes / Yes | Sequencing | 6:80 |
| MDP0000240778 | GTACTGCAAGAAGCACGTCG | CTTCACTCAAAAGCCCATTG | Yes / Yes | Sequencing | 1:50 |
| MDP0000242477 | CTGGCCAAGAAATACCATGC | ACAGCAACTCCCATGCAAAG | No / - | - | - |
| MDP0000244366 | CACAGGAGGCTGGGATAAAA | ATCGACAATGCACAGACCG | Yes / Yes | Sequencing | 1:73 |
| MDP0000245813 | TCACCGGAGTAAAGTCCCAA | TGTTCTCCAAGCAGTCATGG | Yes / Yes | HRM | 3:14 |
| MDP0000255361 | TGTCTGCCACCTCAAGTGAT | TGGCACTCGGAATTTAAACA | No / - | - | - |
| MDP0000255806 | CCAACTGGTGTTGGGAAATC | TCTGCCCTTGCTGTCAGTC | Yes / Yes | HRM | 5:21 |
| MDP0000257364 | CAAGACAAAACCTGTCGGAT | CCGGTATCGGCCATACTCTA | Yes / No | HRM | - |
| MDP0000257814 | GACTGGTGCCCTACTGGGT | ATCATCTCCCTCAGCGGACT | Yes / Yes | HRM | 1:78 |
| MDP0000258088 | AGGAGTTGGCACAGGAAATG | AACTATTGGCATCGACCCAT | No / - | - | - |
| MDP0000258781 | TGGAAGTGGTTTCCCCCT | CCAGGAAATATTGCTCCCAG | Yes / Yes | Sequencing | 1:34 |
| MDP0000266406 | GTGAAATACGACGACCCGTT | CAGATCCATACCCGCCATCT | Yes / No | HRM | - |
| MDP0000271244 | GGGTCGCTATTGAGGGAAA | GTCACTGGCTGCCTTCAAAT | Yes / Yes | HRM | 1:50 |
| MDP0000271291 | GCAGCATGAAGAGAACAAGG | CTCCGCTTCATTGCAGCTT | Yes / Yes | Length size polymorphism | 4:46 |
| MDP0000274009 | GGGTCATGAACGTGGTCTCT | TCATGCGAACCTCCCACT | Yes / Yes | Sequencing | 4:63 |
| MDP0000278207 | CTGCAAGAGCATCCACAACA | TTTTTCTTGGTGACGTCGTT | No / - | - | - |
| MDP0000279221 | ATGAGGAGTGGCCAGAAGAA | ACTCGACCACGAGCCAACT | Yes / Yes | Sequencing | 4:18 |
| MDP0000283542 | AACTAGGCGCGCAGGATT | TTTCTCCAATGCACTTTTCC | Yes / No | Sequencing | - |
| MDP0000284209 | AGGTCCAACTCTGTCGTGCT | CTGCAATCTGATCATCGGC | Yes / No | HRM | - |
| MDP0000284515 | GCTATGTCCTCCGCCACTT | GTCCCTCAGGGTTCAAGGTT | Yes / Yes | Sequencing | 1:50 |
| MDP0000286029 | AAGTGGTCAGGCGTTTCTCT | CATACTCTGGCATTTTCCCA | Yes / Yes | Length size polymorphism | 1:78 |
| MDP0000287300 | TCACTTCCTGGTGGAGCAC | TCCACACCGATATCTGCTCA | Yes / Yes | Sequencing | 5:04 |
| MDP0000288136 | TCATCTGCCTCCTCCACTCT | GCTCGAAGTAGCATCGCC | Yes / Yes | HRM | 4:18 |
| MDP0000290370 | ACAACAATTTGTGCCAGTGC | CCAAAGGGTGGCATTTTTC | Yes / Yes | Sequencing | 3:49 |
| MDP0000290422 | GAATGACCGGAGATGGTGTT | TCAGGACAGCACTGACAATC | Yes / No | HRM | - |
| MDP0000290669 | GTGCACCCTTGTTGCCTT | CCTTCGAAGACCTCGCTGTA | Yes / Yes | Length size polymorphism | 6:56 |
| MDP0000291218 | TCACCAGGCTACCTCACTGG | CAACAGCATCCCAACAACAC | Yes / Yes | Sequencing | 5:42 |
| MDP0000291654 | TCAAAGCGAAAGCCTAGTGG | TAATCGCTCTGCAAATGACC | Yes / No | Sequencing | - |
| MDP0000293008 | CGCCGAGAGGAAGTTCATT | GCTCCGTAAGGGACCTCAGT | Yes / Yes | HRM | 1:73 |
| MDP0000293033 | AGCATGGTTGCTGATGTTCA | GAATGACTGACCGCCTGC | Yes / Yes | HRM | 8:11 |
| MDP0000293986 | GCCCCCTTCTTCCTTACAAA | TTCCTAAACCCAAACGGGAT | Yes / Yes | HRM | 1:14 |
| MDP0000294178 | GAATTTCTCCGATTTCGACG | ACAGTAGGGAAACCCTCGAC | Yes / Yes | HRM | 6:25 |
| MDP0000297803 | GGCTTGTTTACTGCTCAGCC | ACTCGCTCTCATTGTTCCTC | Yes / Yes | Sequencing | 6:45 |
| MDP0000298408 | TTGTTTCTCGTTCCTGGCA | CTTCATTGGTTGCAAAGCTG | Yes / Yes | Sequencing | 8:60 |
| MDP0000299357 | CATCACAGACAGCTTGGAAA | ACTGCATAACCCCTCAAGC | Yes / No | HRM | - |
| MDP0000301367 | GGGACGTTTGCAAATCACTG | CGCCTCACAGCTACCCTCTA | No / - | - | - |
| MDP0000301950 | AATGTCTCCAGGAAATCCCA | CAAATCAATGACCCCCACC | Yes / No | Sequencing | - |
| MDP0000303241 | TGGCAGTTGGGCAGTTTT | GCAAGAGGCCTAAGCTAGTG | Yes / Yes | HRM | 3:49 |
| MDP0000307555 | GATCTGGTGTCCCTGATGCT | GGCCTTGCGTGCTATATCTT | Yes / No | Sequencing | - |
| MDP0000308104 | CCAATTCTCACAAAACCGC | AGTGCACGCAATTCGAAAG | Yes / Yes | HRM | 1:15 |
| MDP0000311522 | CCAACGTGACGAGCAAGAT | GATCAAATAGGGTTGCCTGG | Yes / Yes | HRM | 7:48 |
| MDP0000315021 | TCGACCTCCAGATCAACTCA | TGAGAGCAACAAGGCCACT | Yes / Yes | HRM | 1:14 |
| MDP0000316092 | GCGTTGAGGAAAGAGCCTAT | CAGGGACACCTTTGGTTGC | Yes / Yes | Sequencing | 8:60 |
| MDP0000317051 | CCTTGCAGCTCTGATCTTCC | AGAAAGGCCGTGAAGAATCC | Yes / No | HRM | - |
| MDP0000318208 | GGCCACTTTGATGGGTGAGT | ACAGACCACCATCCTCTTGC | Yes / No | Sequencing | - |
| MDP0000319777 | AATAGTCTTCTTTTGCGGCG | AGCCTGCCTAATATGCTTCA | Yes / Yes | Sequencing | 1:15 |
| MDP0000319817 | TCCTCGATTTGAGGCTCACT | AACAGCTCCAAGTCACCAAT | Yes / Yes | Length size polymorphism | 8:41 |
| MDP0000319973 | CATACCCTTTGGGGACATCA | CCTGTTCACCACCCTTGATT | No / - | - | - |
| MDP0000320356 | GGTCGTGGTGGTTACTATGG | GGTCAATGAAAGGAGTCCCA | Yes / No | HRM | - |
| MDP0000321891 | GCAACATTTGGCAGAGAATG | CTCTCCCTTTGGTCACTGCT | No / - | - | - |
| MDP0000321903 | TGTGCACATGCTGCTGCT | TAACATATGGCAGCGACCAC | Yes / No | HRM | - |
| MDP0000323611 | AACCCCAACAAGAAGATCGG | CCCACATTTAATCCTGGGCT | Yes / Yes | HRM | 7:31 |
| MDP0000324398 | CCTCCCACCAAGACACAAAT | TGGGACATCTCCAACAAGC | No / - | - | - |
| MDP0000370712 | CACGCCAACTTCACCAACTA | GCTGCCCTTCACATTATCG | Yes / Yes | HRM | 7:31 |
| MDP0000375455 | CAACGCTTACGGCTTCTGAT | AAATTTCCTCATCGTGCCCT | Yes / No | HRM | - |
| MDP0000387626 | TGTCGCCAACTGATTCTTCA | GGAGGTTTCTTTCCTCCACA | Yes / Yes | HRM | 1:87 |
| MDP0000398831 | TCCAGATGAGATTGCAAAGC | TCAAAACCACCCTGATTTCT | No / - | - | - |
| MDP0000410984 | AAGGACTACCGCAACATGGA | ATGTAGCCATGTCAGCTCCC | Yes / No | Sequencing | - |
| MDP0000411779 | ACAAATTGTTCTCCTCCCCA | GGATAATGCCCTCTGGCG | Yes / No | HRM | - |
| MDP0000411929 | CTCCTATGGGCCGTGCTC | CTTGTACTCGAAGCTGGGCA | Yes / Yes | HRM | 2:08 |
| MDP0000414898 | GCAAGGGGACATGTTGCT | GGATCCTCCTCGACACCAG | Yes / Yes | HRM | 1:73 |
| MDP0000416706 | GAAGCAGCTGAAAGAGCTGG | TCGGTGAAATCTGCATCAAT | Yes / Yes | HRM | 1:14 |
| MDP0000429824 | GAGCTTCACTTAAAAGGCCA | CATTCTCCAGCCGCCTTCT | No / - | - | - |
| MDP0000448752 | AGACGGGGACTTTGCTGAAC | GGATCAACCCTCACCCATAA | Yes / Yes | HRM | 2:08 |
| MDP0000466557 | CTCCCCCACCGAGCTAAT | CCTGCTCCAAACGGAAGTAG | Yes / No | Sequencing | - |
| MDP0000475073 | CAACGGCAAAGTTGTCCC | CCGATTGTGGTTCCAGAGAC | Yes / Yes | Sequencing | 2:13 |
| MDP0000500656 | ACCTCTTCAAGGATCCAGCA | TCCTCTCCCTTCCCATATCT | Yes / No | HRM | - |
| MDP0000521934 | CGGTTCTGAAGGGGACTTG | CCATTGCTGCTGTTGATGTA | Yes / Yes | HRM | 1:50 |
| MDP0000522218 | CAACTATGCTTTTGGTGCAG | AGTGATTCAACACAGGGTCG | Yes / Yes | HRM | 7:71 |
| MDP0000527695 | AGGCAGGAGGAGAGTGCCTA | GTTTGGTGTGACTGTGTGGC | Yes / Yes | Sequencing | 6:80 |
| MDP0000530903 | AGCAGAGGAGGCACAGGAAG | CAGCACTCATGGCTCTCTCA | Yes / Yes | HRM | 5:08 |
| MDP0000531313 | CCATCGAGTGGCTCCTCC | TATGGTCGGAGGTGGTGGT | No / - | - | - |
| MDP0000536732 | ATGAAATGATGGAGGATGGG | TGAGCTTCATAGCCTCTTGG | No / - | - | - |
| MDP0000545425 | CAGCAGTATACGGCAGCATC | TCTAACAGGGCCTTTTGCAG | Yes / No | Sequencing | - |
| MDP0000547069 | GAATGCCAATGCAGGTAATG | TTGGCGTTTGGGTTAAACAT | Yes / Yes | HRM | 6:80 |
| MDP0000568273 | GCTAGCTGCATCTTTGGGG | GCAATAAGGTGTGTGCAAGC | Yes / Yes | HRM | 6:74 |
| MDP0000580952 | GCTAAGGCATCGAAACATTG | ATCCTTGCCAACCCGAAAT | Yes / No | Sequencing | - |
| MDP0000586151 | TGCTTCATCACCATGCTCTC | GCCTTTCTCTTTCAACCACG | Yes / Yes | HRM | 8:60 |
| MDP0000588193 | CCCTCCTCATTTGGGCTT | AGCTGATGGCATAAAGGTGG | Yes / Yes | HRM | 3:06 |
| MDP0000659511 | ACTGCTCAGGTTTTGTTGGG | AGACAATGGGCCTCCCTATC | Yes / No | Sequencing | - |
| MDP0000661325 | AATGGAGGTGCAGAACCTAA | CTCTTCGGAAACCTCCTTCC | Yes / Yes | HRM | 2:28 |
| MDP0000661533 | TACGACTGCGTTTCATCCC | CATCTCCACACTGCGAACAG | Yes / Yes | Sequencing | 5:04 |
| MDP0000661960 | AGGAGTTCCATACCCCAAGC | CGGGATTTATCGGTGCAGTA | Yes / No | Sequencing | - |
| MDP0000680183 | ACCCTTTGATGGACTGGGTT | ATCTCAAAGCAACCTCTGGC | Yes / No | HRM | - |
| MDP0000683905 | CTCACTCACCGGAAATCGTT | AGATCAGGCTTCTTCCTCGG | No / - | - | - |
| MDP0000690031 | TCCTCAAGTTTCAAATCGCA | ACTGATGCGCCAGTCTTTTG | No / - | - | - |
| MDP0000709066 | GACGACTCAGAGCCGCCTA | CTTCATGAGTTGGCTCCTCG | No / - | - | - |
| MDP0000713493 | GAACACCTTCCTGCAGTACC | CTCCATTAAATCCTGACGGC | No / - | - | - |
| MDP0000722943 | CAACAGCTCCATCATCCTCA | CAGGGGTTGAGCATTGGATA | Yes / Yes | HRM | 2:28 |
| MDP0000734607 | GGAAGTTGGCTTCAACTGGA | CACAAGCCTTAAAGACCCTG | Yes / No | HRM | - |
| MDP0000778016 | CAGGATTTCTGCAGACAGGT | GGCAATCTCAACAGGGAAAC | Yes / No | HRM | - |
| MDP0000788934 | CACTTTGCCCTCAGCCAG | GGAGCGGCTTAAGGATGAT | Yes / Yes | HRM | 5:21 |
| MDP0000794663 | GTTACATCTGTGTTCCGGGC | AGAGATGGAAGCTGCAAGGA | Yes / Yes | Sequencing | 5:46 |
| MDP0000821787 | GTGATGGAGGGGCTACCG | AATCCTCAACAACGACGCC | Yes / No | Sequencing | - |
| MDP0000822588 | TTTCTCCTTCAGACCTGCTC | TTTGCATTGACTTTAGGCCA | Yes / Yes | HRM | 6:65 |
| MDP0000828015 | TCTGCACCCAAACCAAACTT | CCGACTCCAAGCTCACATAA | Yes / Yes | HRM | 3:49 |
| MDP0000849292 | ACTTCTCCCGCCTCTCTCTC | TCTGAGTAGCCATTCTTGGG | Yes / Yes | HRM | 1:14 |
| MDP0000877582 | CTCACGCAAGACCTTCATGG | ACTCCATGGTGGTTCACTGG | Yes / Yes | HRM | 1:78 |
| MDP0000936748 | GCAAAGTGGCCAATGTCAA | TGGTGTGTTGCTGCTCTTTC | Yes / Yes | HRM | 4:18 |
| MDP0000950533 | TTGGCTGCATGAAAGTGTTG | CCCCTACAGGTTGCAGTCAA | Yes / No | HRM | - |
