# Supplementary material for: Comparative analysis of rosaceous genomes and the reconstruction of a putative ancestral genome for the family
Source: BMC Evol Biol. 2011 Jan 12;11:9. doi: 10.1186/1471-2148-11-9 (PMC3033827; doi:10.1186/1471-2148-11-9)
Supplement: Additional file 2 — Table S2, Fragaria genetic markers. Table S2 lists the 237 genetic markers of the diploid Fragaria FV×FN reference map, along with their marker type, method of detection and the mapping bins to which they are located. [file 1471-2148-11-9-S2.DOC]

**Table S2.** The 237 genetic markers of the diploid Fragaria FV×FN

reference map, along with their marker type, method of detection

**and the mapping bins to which they are located.**

| **Marker name** | **Marker type** | **Detection method** | **Bin FV×FN** |
| --- | --- | --- | --- |
| AC10 | RFLP | RFLP | IV:46 |
| AC24 | RFLP | RFLP | IV:26 |
| AC31 | RFLP | RFLP | VII:21 |
| AC32 | RFLP | RFLP | IV:26 |
| AC44 | RFLP | RFLP | II:75 |
| AC49 | RFLP | RFLP | V:15 |
| AC50 | RFLP | RFLP | III:44 |
| AC8 | RFLP | RFLP | I:90 |
| ACL5 | Gene specific | Sequencing | II:50 |
| ACO | Gene specific | Length polymorphism | VI:87 |
| ADH | Gene specific | Length polymorphism | II:29 |
| AG102 | RFLP | RFLP | VI:68 |
| AG104 | RFLP | RFLP | VI:68 |
| AG106 | RFLP | RFLP | VI:115 |
| AG33 | RFLP | RFLP | V:73 |
| AG35 | RFLP | RFLP | VII:21 |
| AG47 | RFLP | RFLP | II:29 |
| AG53 | RFLP | RFLP | IV:26 |
| AG56 | RFLP | RFLP | VI:68 |
| AG6 | RFLP | RFLP | III:44 |
| AMY | Gene specific | Sequencing | II:56 |
| ANS | Gene specific | Length polymorphism | V:15 |
| APB | Gene specific | Length polymorphism | II:08 |
| APX | Gene specific | Length polymorphism | III:53 |
| BCS | Gene specific | Length polymorphism | VII:21 |
| BFACT002 | SSR | Length polymorphism | II:29 |
| CC116 | RFLP | RFLP | VI:07 |
| CC2 | RFLP | RFLP | VI:31 |
| CC47 | RFLP | RFLP | III:13 |
| CC8 | RFLP | RFLP | VI:87 |
| CEL-1 | Gene specific | Length polymorphism | IV:68 |
| CEL-2 | Gene specific | Length polymorphism | V:29 |
| CFACT080 | SSR | Length polymorphism | IV:26 |
| CFACT091 | SSR | Length polymorphism | V:46 |
| CFACT152 | SSR | Length polymorphism | VI:68 |
| CFACT157 | SSR | Length polymorphism | VII:21 |
| CFACT159 | SSR | Length polymorphism | II:47 |
| CFACT168 | SSR | Length polymorphism | III:28 |
| CFVCT003 | SSR | Length polymorphism | V:73 |
| CFVCT016 | SSR | Length polymorphism | V:48 |
| CFVCT030 | SSR | Length polymorphism | VI:68 |
| CHI | Gene specific | Length polymorphism | VII:21 |
| CHS | Gene specific | Length polymorphism | VII:21 |
| CPK | Gene specific | Sequencing | III:13 |
| DFR | Gene specific | Length polymorphism | II:75 |
| EFaTR1976 | EST | Sequencing | II:75 |
| EFaUF7084 | EST | Sequencing | VII:64 |
| EFaUF7248 | EST | Sequencing | II:75 |
| EFaUF7699 | EST | Sequencing | IV:26 |
| EFvNH8484 | EST | Sequencing | VII:40 |
| EFvNH8894 | EST | Sequencing | II:75 |
| EFvNH9257 | EST | Sequencing | VII:21 |
| EFvNH9852 | EST | Sequencing | VII:40 |
| EFvUF6868 | EST | Sequencing | II:75 |
| EFvVB1231 | EST | Sequencing | IV:26 |
| EFvVB1923 | EST | Sequencing | VI:68 |
| EFvVB2013 | EST | Sequencing | V:73 |
| EFvVB2119 | EST | Sequencing | VI:31 |
| EFvVB2179 | EST | Sequencing | IV:26 |
| EKO | Gene specific | Length polymorphism | II:00 |
| EMFn111 | SSR | Length polymorphism | IV:46 |
| EMFn119 | SSR | Length polymorphism | VI:68 |
| EMFn128 | SSR | Length polymorphism | I:74 |
| EMFn134 | SSR | Length polymorphism | II:50 |
| EMFn152 | SSR | Length polymorphism | I:74 |
| EMFn153 | SSR | Length polymorphism | VI:87 |
| EMFn181 | SSR | Length polymorphism | V:73 |
| EMFn207 | SSR | Length polymorphism | III:57 |
| EMFn213 | SSR | Length polymorphism | VII:40 |
| EMFv022 | SSR | Length polymorphism | II:29 |
| EMFv111 | SSR | Length polymorphism | V:73 |
| EMFv132 | SSR | Length polymorphism | IV:46 |
| EMFvi038 | SSR | Length polymorphism | III:53 |
| EPpCU1785 | EST | Sequencing | I:45 |
| EPpCU1830 | EST | Sequencing | VI:14 |
| EPpCU2875 | EST | Sequencing | VII:21 |
| EPpCU7308 | EST | Sequencing | II:47 |
| EPpCU9223 | EST | Sequencing | VII:45 |
| EPpCU9257 | EST | Sequencing | VII:40 |
| EPpCU9642 | EST | Sequencing | II:29 |
| EPpCU9910 | EST | Sequencing | I:08 |
| F3H | Gene specific | Length polymorphism | I:41 |
| FG215 | RFLP | RFLP | III:44 |
| FG3 | RFLP | RFLP | III:44 |
| FG36 | RFLP | RFLP | II:47 |
| FG37 | RFLP | RFLP | II:29 |
| FPS | Gene specific | Sequencing | III:13 |
| GP | Gene specific | Sequencing | V:21 |
| LOX | Gene specific | Length polymorphism | IV:46 |
| LY29 | RFLP | RFLP | II:14 |
| MC225 | RFLP | RFLP | VI:68 |
| MC45 | RFLP | RFLP | VII:89 |
| MEX | Gene specific | Sequencing | VII:90 |
| Omt1 | RFLP | RFLP | VII:70 |
| PAO | Gene specific | Sequencing | VII:40 |
| PC1 | RFLP | RFLP | III:44 |
| PC101 | RFLP | RFLP | II:14 |
| PC12 | RFLP | RFLP | VI:115 |
| PC14 | RFLP | RFLP | V:15 |
| PC21 | RFLP | RFLP | I:47 |
| PC78 | RFLP | RFLP | IV:46 |
| PES | Gene specific | Sequencing | I:21 |
| PGLM | Gene specific | Length polymorphism | VI:31 |
| PL | Gene specific | Length polymorphism | V:37 |
| PRU1 | RFLP | RFLP | II:29 |
| QR | Gene specific | Length polymorphism | VI:115 |
| ROSCOS 0052 | RosCOS | Sequencing | II:14 |
| ROSCOS 0058 | RosCOS | Sequencing | VI:07 |
| ROSCOS 0470 | RosCOS | Sequencing | III:53 |
| ROSCOS 0509 | RosCOS | Sequencing | III:53 |
| ROSCOS 0510 | RosCOS | Sequencing | V:81 |
| ROSCOS 0517 | RosCOS | Sequencing | II:08 |
| ROSCOS 0530 | RosCOS | Sequencing | II:08 |
| ROSCOS 0536 | RosCOS | Sequencing | V:48 |
| ROSCOS 0537 | RosCOS | Sequencing | II:47 |
| ROSCOS 0541 | RosCOS | Sequencing | VII:21 |
| ROSCOS 0546 | RosCOS | Sequencing | III:44 |
| ROSCOS 0547 | RosCOS | Sequencing | VI:68 |
| ROSCOS 0601 | RosCOS | Sequencing | II:50 |
| ROSCOS 0602 | RosCOS | Sequencing | VII:21 |
| ROSCOS 0645 | RosCOS | Sequencing | III:13 |
| ROSCOS 0784 | RosCOS | Sequencing | II:29 |
| ROSCOS 0955 | RosCOS | Sequencing | III:53 |
| ROSCOS 1097 | RosCOS | Sequencing | VI:72 |
| ROSCOS 1098 | RosCOS | Sequencing | VI:70 |
| ROSCOS 1101 | RosCOS | Sequencing | IV:80 |
| ROSCOS 1113 | RosCOS | Sequencing | III:57 |
| ROSCOS 1125 | RosCOS | Sequencing | II:47 |
| ROSCOS 1139 | RosCOS | Sequencing | V:73 |
| ROSCOS 1140 | RosCOS | Sequencing | VI:68 |
| ROSCOS 1157 | RosCOS | Sequencing | VII:40 |
| ROSCOS 1160 | RosCOS | Sequencing | V:46 |
| ROSCOS 1167 | RosCOS | Sequencing | V:73 |
| ROSCOS 1170 | RosCOS | Sequencing | VI:68 |
| ROSCOS 1205 | RosCOS | Sequencing | V:46 |
| ROSCOS 1217 | RosCOS | Sequencing | IV:68 |
| ROSCOS 1222 | RosCOS | Sequencing | II:08 |
| ROSCOS 1231 | RosCOS | Sequencing | IV:20 |
| ROSCOS 1234 | RosCOS | Sequencing | VI:14 |
| ROSCOS 1247 | RosCOS | Sequencing | VI:68 |
| ROSCOS 1248 | RosCOS | Sequencing | II:14 |
| ROSCOS 1255 | RosCOS | Sequencing | V:73 |
| ROSCOS 1257 | RosCOS | Sequencing | V:73 |
| ROSCOS 1259 | RosCOS | Sequencing | III:57 |
| ROSCOS 1261 | RosCOS | Sequencing | V:73 |
| ROSCOS 1271 | RosCOS | Sequencing | V:37 |
| ROSCOS 1272 | RosCOS | Sequencing | II:14 |
| ROSCOS 1276 | RosCOS | Sequencing | VII:21 |
| ROSCOS 1278 | RosCOS | Sequencing | II:29 |
| ROSCOS 1281 | RosCOS | Sequencing | VII:21 |
| ROSCOS 1289 | RosCOS | Sequencing | II:14 |
| ROSCOS 1291 | RosCOS | Sequencing | VII:64 |
| ROSCOS 1295 | RosCOS | Sequencing | VI:68 |
| ROSCOS 1315 | RosCOS | Sequencing | II:00 |
| ROSCOS 1326 | RosCOS | Sequencing | I:21 |
| ROSCOS 1335 | RosCOS | Sequencing | II:47 |
| ROSCOS 1338 | RosCOS | Sequencing | II:14 |
| ROSCOS 1341 | RosCOS | Sequencing | II:29 |
| ROSCOS 1354 | RosCOS | Sequencing | VI:07 |
| ROSCOS 1360 | RosCOS | Sequencing | VI:68 |
| ROSCOS 1363 | RosCOS | Sequencing | III:53 |
| ROSCOS 1367 | RosCOS | Sequencing | VI:31 |
| ROSCOS 1378 | RosCOS | Sequencing | V:73 |
| ROSCOS 1381 | RosCOS | Sequencing | III:13 |
| ROSCOS 1384 | RosCOS | Sequencing | III:17 |
| ROSCOS 1396 | RosCOS | Sequencing | I:41 |
| ROSCOS 1407 | RosCOS | Sequencing | IV:20 |
| ROSCOS 1408 | RosCOS | Sequencing | VII:21 |
| ROSCOS 1410 | RosCOS | Sequencing | V:73 |
| ROSCOS 1412 | RosCOS | Sequencing | VI:68 |
| ROSCOS 1428 | RosCOS | Sequencing | II:47 |
| ROSCOS 1435 | RosCOS | Sequencing | V:37 |
| ROSCOS 1459 | RosCOS | Sequencing | IV:26 |
| ROSCOS 1482 | RosCOS | Sequencing | V:48 |
| ROSCOS 1509 | RosCOS | Sequencing | III:53 |
| ROSCOS 1511 | RosCOS | Sequencing | II:14 |
| ROSCOS 1519 | RosCOS | Sequencing | VI:07 |
| ROSCOS 1520 | RosCOS | Sequencing | II:56 |
| ROSCOS 1527 | RosCOS | Sequencing | IV:26 |
| ROSCOS 1532 | RosCOS | Sequencing | V:46 |
| ROSCOS 1537 | RosCOS | Sequencing | II:00 |
| ROSCOS 1538 | RosCOS | Sequencing | I:08 |
| ROSCOS 1549 | RosCOS | Sequencing | II:56 |
| ROSCOS 1602 | RosCOS | Sequencing | IV:20 |
| ROSCOS 1616 | RosCOS | Sequencing | V:73 |
| ROSCOS 1617 | RosCOS | Sequencing | VI:14 |
| ROSCOS 1623 | RosCOS | Sequencing | I:90 |
| ROSCOS 1629 | RosCOS | Sequencing | V:37 |
| ROSCOS 1635 | RosCOS | Sequencing | I:90 |
| ROSCOS 1659 | RosCOS | Sequencing | VI:14 |
| ROSCOS 1674 | RosCOS | Sequencing | VI:68 |
| ROSCOS 1814 | RosCOS | Sequencing | V:73 |
| ROSCOS 1829 | RosCOS | Sequencing | V:46 |
| ROSCOS 1983 | RosCOS | Sequencing | I:90 |
| ROSCOS 2040 | RosCOS | Sequencing | III:53 |
| ROSCOS 2050 | RosCOS | Sequencing | VII:21 |
| ROSCOS 2054 | RosCOS | Sequencing | II:29 |
| ROSCOS 2105 | RosCOS | Sequencing | I:90 |
| ROSCOS 2118 | RosCOS | Sequencing | II:29 |
| ROSCOS 2134 | RosCOS | Sequencing | II:08 |
| ROSCOS 2343 | RosCOS | Sequencing | I:21 |
| ROSCOS 2346 | RosCOS | Sequencing | VII:21 |
| ROSCOS 2364 | RosCOS | Sequencing | I:41 |
| ROSCOS 2372 | RosCOS | Sequencing | V:37 |
| ROSCOS 2409 | RosCOS | Sequencing | III:53 |
| ROSCOS 2411 | RosCOS | Sequencing | V:73 |
| ROSCOS 2573 | RosCOS | Sequencing | VII:40 |
| ROSCOS 2581 | RosCOS | Sequencing | III:44 |
| ROSCOS 2591 | RosCOS | Sequencing | V:48 |
| ROSCOS 2592 | RosCOS | Sequencing | III:53 |
| ROSCOS 2596 | RosCOS | Sequencing | II:29 |
| ROSCOS 2601 | RosCOS | Sequencing | IV:20 |
| ROSCOS 2603 | RosCOS | Sequencing | III:53 |
| ROSCOS 2617 | RosCOS | Sequencing | III:57 |
| ROSCOS 2623 | RosCOS | Sequencing | VII:21 |
| ROSCOS 2624 | RosCOS | Sequencing | III:13 |
| ROSCOS 2626 | RosCOS | Sequencing | VI:68 |
| ROSCOS 2892 | RosCOS | Sequencing | V:48 |
| ROSCOS 2899 | RosCOS | Sequencing | III:13 |
| ROSCOS 2970 | RosCOS | Sequencing | VI:115 |
| ROSCOS 3055 | RosCOS | Sequencing | V:37 |
| ROSCOS 3524 | RosCOS | Sequencing | IV:26 |
| ROSCOS 3613 | RosCOS | Sequencing | IV:68 |
| ROSCOS 3695 | RosCOS | Sequencing | V:46 |
| ROSCOS 3702 | RosCOS | Sequencing | V:37 |
| ROSCOS 3710 | RosCOS | Sequencing | V:15 |
| ROSCOS 3742 | RosCOS | Sequencing | II:14 |
| ROSCOS 3749 | RosCOS | Sequencing | V:37 |
| ROSCOS 3767 | RosCOS | Sequencing | VI:72 |
| ROSCOS 3781 | RosCOS | Sequencing | II:29 |
| ROSCOS 3782 | RosCOS | Sequencing | V:73 |
| ROSCOS 3783 | RosCOS | Sequencing | V:73 |
| SIP | Gene specific | Sequencing | VI:43 |
| TSA3 | RFLP | RFLP | I:41 |
| UDF016 | SSR | Length polymorphism | III:28 |
| UFFxa08C11 | SSR | Length polymorphism | II:29 |
| ZIP | Gene specific | Length polymorphism | VI:72 |
