# Supplementary material for: Comparative analysis of rosaceous genomes and the reconstruction of a putative ancestral genome for the family
Source: BMC Evol Biol. 2011 Jan 12;11:9. doi: 10.1186/1471-2148-11-9 (PMC3033827; doi:10.1186/1471-2148-11-9)
Supplement: Additional file 3 — Table S3, markers used in this work. Table S3 lists the number and type of markers used in this work. All the available markers were tested against Malus × domestica genome using GMAP and BLASTN. Syntenic markers are those that fulfilled the criteria defined for conservation of synteny in the Materials and Methods section. [file 1471-2148-11-9-S3.DOC]

**Table S3. Markers used in this work. All the available markers were tested against *Malus × domestica* genome using GMAP and BlastN. Syntenic markers are those that fulfilled the criteria defined for synteny in the Materials and Methods section.**

| **Marker type** | **Total investigated** | **GMAP/Blast** | **Syntenic** | **Mapped in Prunus** | **Mapped in Fragaria** | **Reference** |
| --- | --- | --- | --- | --- | --- | --- |
| SSR | 572 | 214 | 119 | 105 | 14 | Dirlewanger et al., 2004; Howad et al., 2005 |
| RFLP | 117 | 72 | 49 | 49 | 19 | Dirlewanger et al., 2004; Vilanova et al., 2008 |
| EST/CG | 235 | 182 | 141 | 133 | 25 | Sargent et al., 2009 ; Illa et al., unpublished data |
| RosCOS | 549 | 545 | 439 | 439 | 90 | Cabrera et al., 2009 |
| New *Prunus* |  | 94 | 58 | 58 |  | present work |
| **Total** | 1473 | 1107 | 806 | 784 | 148 |  |
