# Supplementary figures and images for: Comparative analysis of rosaceous genomes and the reconstruction of a putative ancestral genome for the family
Source: BMC Evol Biol. 2011 Jan 12;11:9. doi: 10.1186/1471-2148-11-9 (PMC3033827; doi:10.1186/1471-2148-11-9)

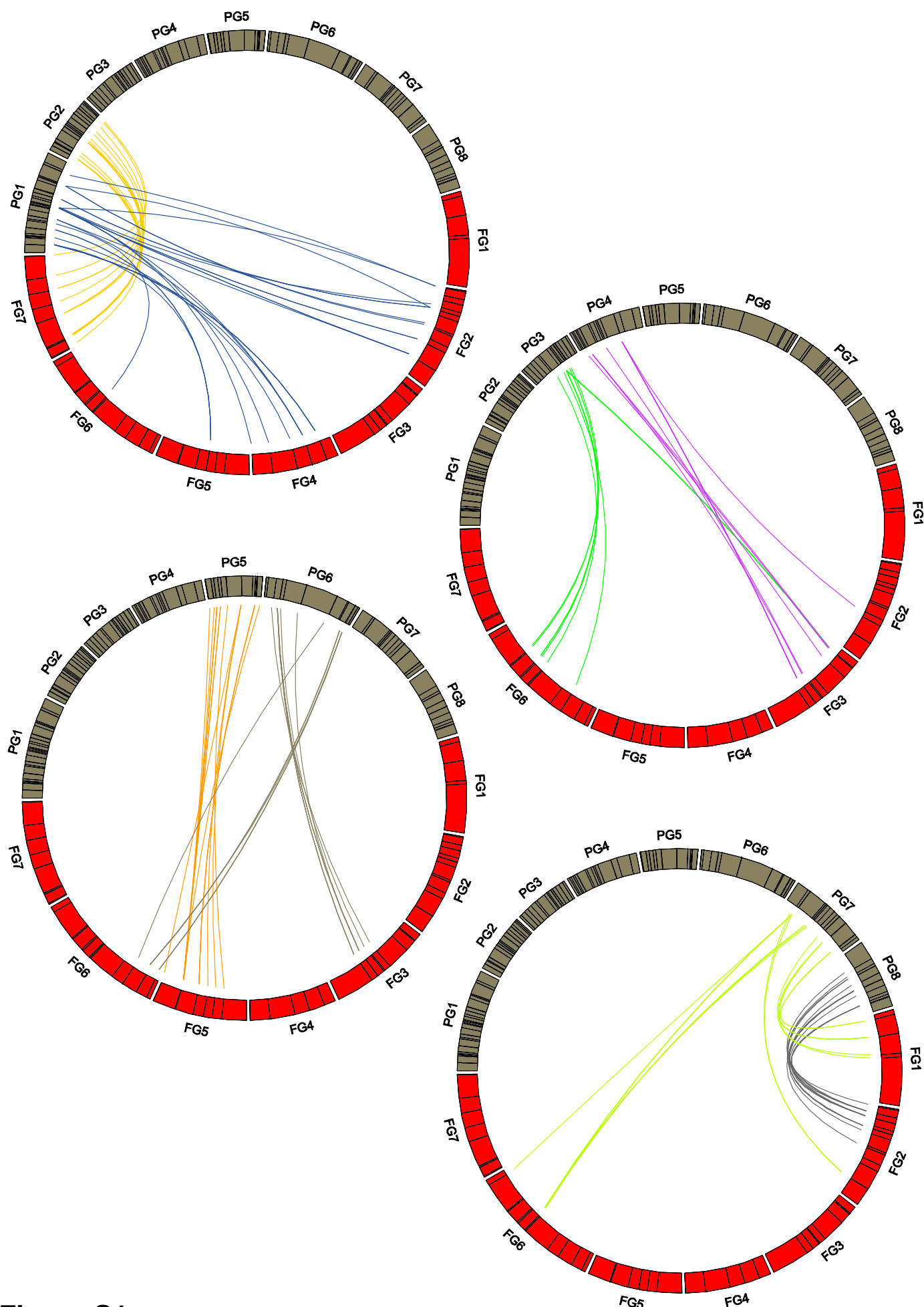

Figure S1

Supplement: Additional file 4 — Figure S1, Prunus and Fragaria map comparison. Figure S1 shows the comparative analysis between Prunus (PG1 to PG8) and diploid Fragaria (FG1 to FG7) reference map using the Circos program. Only markers that were also present in the Malus genome sequences were included in the Fragaria-Prunus comparison. [file 1471-2148-11-9-S4.PDF]

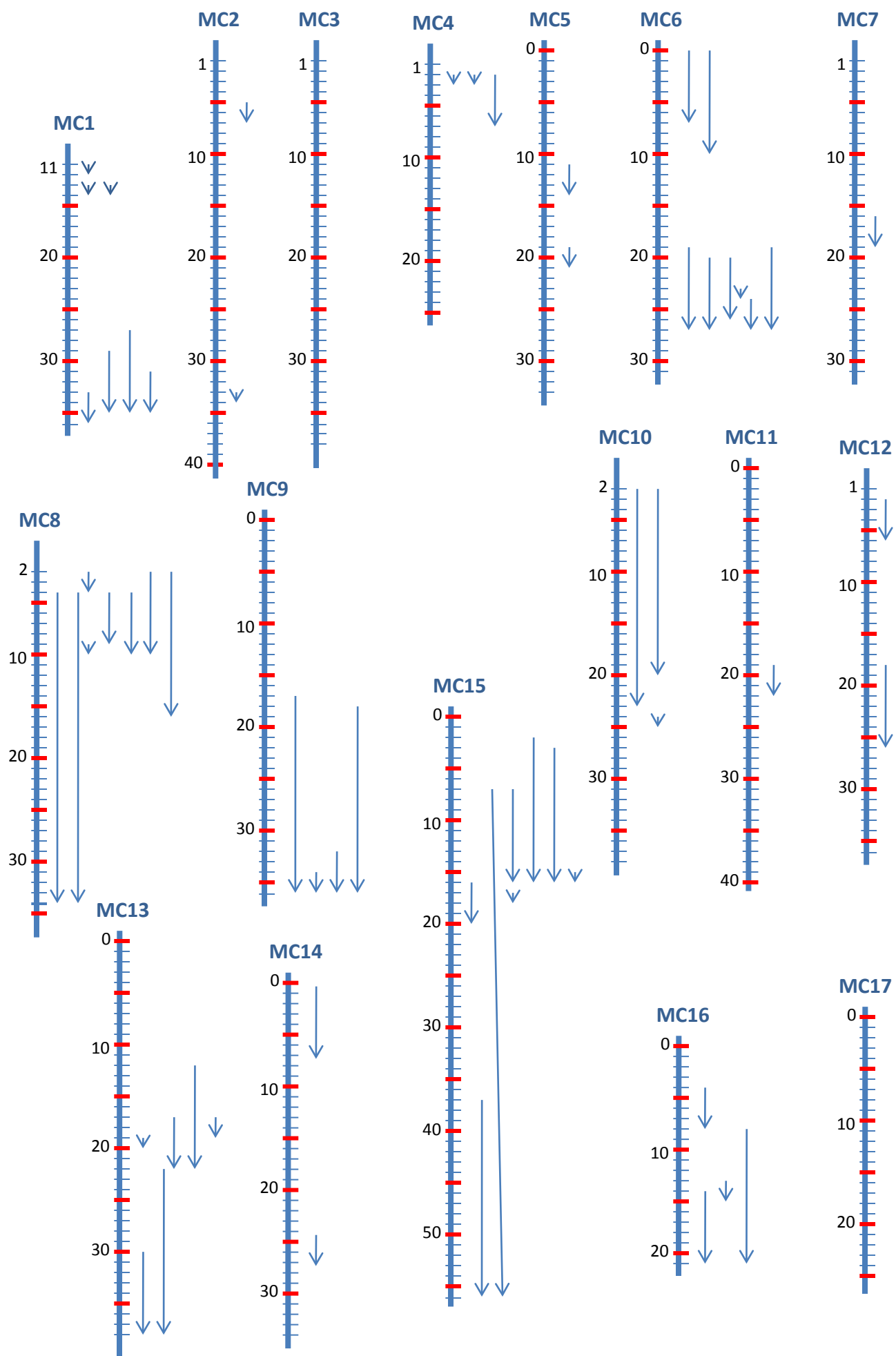

Figure S2

Supplement: Additional file 5 — Figure S2, Malus and Prunus inversions in the Malus genome. Figure S2 shows the positions in the Malus genome of the inversions detected between Malus and Prunus using the complete set of 784 anchor markers in Prunus. Apple chromosomes are divided in fractions of 10 Mb. Arrows indicate the presence of a predicted inversion. [file 1471-2148-11-9-S5.PDF]
